# Decreasing incidence of hepatocellular carcinoma among most racial groups: SEER‐22, 2000–2019

**DOI:** 10.1002/cam4.6537

**Published:** 2023-09-30

**Authors:** Thomas R. O'Brien, Susan S. Devesa, Jill Koshiol, Jorge A. Marrero, Meredith S. Shiels

**Affiliations:** ^1^ Infections and Immunoepidemiology Branch, Division of Cancer Epidemiology and Genetics National Cancer Institute Bethesda Maryland USA; ^2^ Division of Gastroenterology and Hepatology Perelman School of Medicine at the University of Pennsylvania Philadelphia Pennsylvania USA

**Keywords:** epidemiology, health disparities, liver cancer, surveillance

## Abstract

**Background:**

Hepatocellular carcinoma (HCC) incidence was rising in the United States. Previously, using data collected by the Surveillance, Epidemiology, and End Results (SEER) Program through 2017, we found that overall incidence had begun to decline, although not in Black and American Indian/Alaska Native (AI/AN) populations. Utilizing expanded SEER data encompassing ~50% of the population, we examined secular trends and demographic differences in HCC incidence through 2019.

**Methods:**

We included cases of HCC diagnosed in adults aged ≥20 years residing in SEER‐22 registry areas. We examined case counts, incidence rates (per 100,000 person‐years), annual percent changes (APCs), and calendar years when APCs changed significantly.

**Results:**

HCC incidence increased from 5.56 in 2000 to 8.89 in 2009 (APC, 5.17%), then rose more slowly during 2009–2015 (APC, 2.28%). After peaking at 10.03 in 2015, incidence fell to 9.20 in 2019 (APC, −2.26%). In Asian/Pacific Islanders (A/PI), the decline began in 2007 and accelerated in 2015 (APCs: 2007–2015, −1.84%; 2015–2019, −5.80%). In 2014, incidence began to fall in the White (APC: 2014–2019, −1.11%) and Hispanic populations (APC: 2014–2019, −1.72%). In 2016, rates began to fall in Black individuals (APC: 2016–2019, −6.05%). In the AI/AN population, incidence was highest in 2017, although the subsequent decline was not statistically significant. In 2019, population‐specific rates were: White, 6.94; Black, 10.74; A/PI, 12.11; AI/AN, 14.56; Hispanic, 15.48.

**Conclusion:**

HCC incidence is now decreasing in most US racial/ethnic populations, including among Black individuals. The onset of decline differed among racial/ethnic groups and wide disparities in HCC rates remain.

## 1 INTRODUCTION

Liver cancer is a highly lethal malignancy that is the sixth leading cause of cancer mortality in the United States.^1^ The incidence of hepatocellular carcinoma (HCC), the most common form of liver cancer, rose sharply in the United States beginning in ~1980;^2^ however, using data from 21 Surveillance, Epidemiology, and End Results (SEER) Program registries that was available through calendar year 2017, we previously found that overall HCC incidence peaked in 2014–2015.^3^, ^4^ We also observed marked differences in secular trends among racial/ethnic groups with an early decline in HCC incidence among Asian/Pacific Islander individuals, but no evidence of a decline in Black or American Indian/Alaska Native populations.^3^, ^4^

Recently, the SEER Program was modified and expanded to encompass 22 registries (SEER‐22), which together capture data for almost 50% of the US population. With this larger dataset, we have now examined temporal trends and demographic differences in HCC incidence through 2019 to provide additional information regarding racial disparities for this cancer.

## 2 MATERIALS AND METHODS

### 2.1 Data source

The National Cancer Institute funds population‐based SEER Program registries around the United States. For all newly diagnosed cancers among residents of those catchment areas, information is collected on patient demographics, date of diagnosis, and tumor characteristics, but not potential etiologic factors (https://seer.cancer.gov/about/overview.html). For the present study, we included HCC cases diagnosed between 2000 and 2019 among adults aged ≥20 years residing in 22 SEER registries (Alaska Native Tumor Registry, Connecticut, Atlanta, Greater Georgia, Rural Georgia, San Francisco‐Oakland, San Jose‐Monterey, Los Angeles, Greater California, Hawaii, Iowa, Idaho, Illinois, Kentucky, Louisiana, Massachusetts, New Mexico, New Jersey, New York, Seattle‐Puget Sound, Texas, and Utah; https://seer.cancer.gov/registries/terms.html accessed October 27, 2022).

### 2.2 Case definitions and other variables

We selected cancers for analysis based on codes from the International Classification of Diseases for Oncology, Third Edition [ICD‐O‐3]). We defined HCC as cancers with ICD‐O‐3 primary site C220 (liver) and histology codes 8170–8175 (hepatocellular carcinoma). We examined incidence in five nonoverlapping categories for race/ethnicity (White; Black; Asian/Pacific Islander; American Indian/Alaska Native; Hispanic) All individuals of Hispanic ethnicity were classified as ‘Hispanic’ and excluded from other race/ethnicity categories.

### 2.3 Statistical analysis

We examined the trends in overall HCC incidence and also stratified these analyses by sex, age at diagnosis (20–39; 40–64; 65–79; ≥80 years), race/ethnicity and SEER registry. Rates among American Indian/Alaska Native individuals were restricted to the Purchase/Referred Delivery Areas.

We calculated case counts and incidence rates (per 100,000 person‐years) using SEER*Stat (version 8.3.6), age standardizing the rates to the 2000 US standard population by 5‐year age groups.

To quantify trends in incidence, we used the Joinpoint Regression Analysis program (version 4.7.0.0) to calculate annual percent changes (APCs) and corresponding 95% confidence intervals (CIs). This program selects the best fitting log‐linear regression model to identify calendar years during which the APCs changed significantly. We used 2‐sided *t*‐tests to calculate *p*‐values.

## 3 RESULTS

During 2000–2019, a total of 194,371 HCC cases were diagnosed among residents of SEER‐22 registry catchment areas aged ≥20 years. HCC rates varied markedly by demographic characteristics (Table 1). The age‐standardized incidence rate (per 100,000 person‐years) among men was ~4 times that among women (14.28 vs. 3.80). The highest incidence was in those aged 65–74 years (25.80), followed by those >75 years (23.82), and those 55–64 years (20.92), all much higher than in those aged 20–54 years (2.40). Overall HCC rates also differed widely by race/ethnicity: 6.24 in White individuals, 11.23 in Black individuals, 15.43 in Hispanic individuals, 15.66 in Asian/Pacific Islanders, and 17.05 in the American Indian/Alaska Native population. HCC rates varied notably by cancer registry, from a high of 13.67 among Alaska Natives to about 11 at several regions (Texas, Hawaii, San Francisco‐Oakland SMSA, and San Jose‐Monterey), 8.51 in New York, and a low of 5.22 in Iowa (Table 1).

**TABLE 1:** Incidence (per 100,000 persons) of hepatocellular carcinoma, by sex, age, and race/ethnicity—SEER 22, 2000–2019. Individuals of Hispanic ethnicity were classified as ‘Hispanic’ and excluded from other race/ethnicity categories.

	Cases	Rate
Overall	194,371	8.64
Sex		
Male	148,664	14.28
Female	45,707	3.80
Age (years)		
20–54 years	35,525	2.40
55–64 years	67,204	20.92
65–74 years	52,384	25.80
75+ years	39,258	23.82
Race/ethnicity		
White (non‐Hispanic)	97,088	6.24
Black (non‐Hispanic)	25,923	11.23
Hispanic (All Races)	44,752	15.43
Asian or Pacific Islander (non‐Hispanic)	24,523	15.66
American Indian/Alaska Native (non‐Hispanic)	1188	17.05
Registry (ordered by descending incidence)		
Alaska Natives	166	13.67
San Jose‐Monterey (SJM/)	4067	11.26
San Francisco‐Oakland SMSA (SF)	7806	11.12
Hawaii	2530	11.04
Texas	37,922	10.95
Los Angeles (LA)	13,598	9.96
Seattle (Puget Sound)	6887	9.42
California (excluding SF/SJM/LA)	28,346	9.42
New Mexico	3017	9.16
Louisiana	6631	9.14
New York	27,096	8.51
Massachusetts	8439	7.63
Atlanta (Metropolitan)	3265	7.57
New Jersey	10,198	7.01
Connecticut	4267	6.97
Illinois	13,264	6.66
Greater Georgia	6193	6.60
Kentucky	4689	6.54
Idaho	1393	5.75
Rural Georgia	137	5.63
Utah	1728	5.36
Iowa	2732	5.22

**FIGURE 1:**
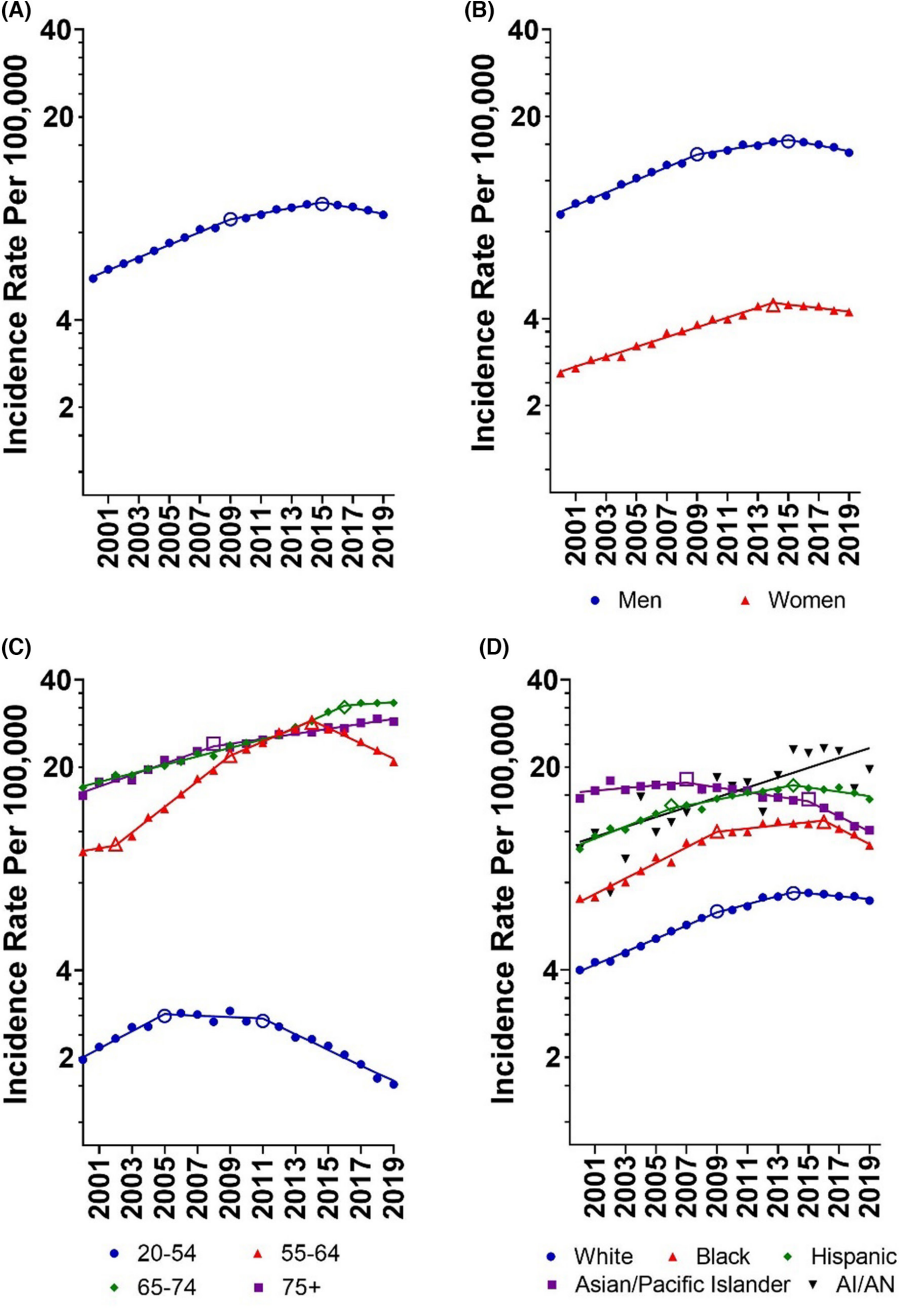
Age‐adjusted rates (per 100,000 persons) of hepatocellular carcinoma in the United States, overall (A) as well as by sex (B), age group (C), and race/ethnicity (D)—SEER 22, 2000–2019. Individuals of Hispanic ethnicity were classified as ‘Hispanic’ and excluded from other race/ethnicity categories. Open symbols indicate a joinpoint where the annual percent change differs between segments (*p* < 0.05).

Overall HCC rates rose and then fell during the 2000–2019 study period (Figure 1A; Table 2). Using output from the Joinpoint Regression Analysis program to identify calendar years when an APC changed significantly, we found that HCC Incidence increased from 5.56 in 2000 to 8.89 in 2009 for an annual percent change (APC) of 5.18% (95% CI: 4.78 to 5.57) and then rose more slowly to 10.03 in 2015 (APC: 2.27%; 95% CI: 1.55 to 3.01). After reaching that peak, HCC rates declined consistently and significantly, falling to 9.20 by 2019, (APC: −2.25%; 95% CI: −3.17 to −1.33).

**TABLE 2:** Annual percent changes (APCs) in the incidence of hepatocellular carcinoma overall and by sex, age, and race/ethnicity—SEER 22, 2000–2019. Individuals of Hispanic ethnicity were classified as ‘Hispanic’ and excluded from other race/ethnicity categories.

	Segment start	Segment end	APC	95% LCL	95% UCL	*p*‐Value
Overall	2000	2009	5.18	4.78	5.57	<0.001
	2009	2015	2.27	1.55	3.00	<0.001
	2015	2019	−2.25	−3.17	−1.33	<0.001
Male	2000	2009	5.18	4.63	5.73	<0.001
	2009	2015	1.99	0.99	3.00	0.001
	2015	2019	−2.26	−3.52	−0.97	0.002
Female	2000	2014	4.00	3.71	4.29	<0.001
	2014	2019	−1.40	−2.44	−0.35	0.013
20–54 years	2000	2005	6.86	4.32	9.47	<0.001
	2005	2011	−0.57	−2.61	1.51	0.558
	2011	2019	−6.02	−7.13	−4.90	<0.001
55–64 years	2000	2002	2.25	−9.26	15.22	0.683
	2002	2009	10.67	9.03	12.35	<0.001
	2009	2014	5.75	3.70	7.85	<0.001
	2014	2019	−5.86	−7.11	−4.59	<0.001
65–74 years	2000	2012	3.43	3.11	3.75	<0.001
	2012	2016	6.02	4.01	8.07	<0.001
	2016	2019	0.91	−0.74	2.60	0.254
75+ years	2000	2008	4.64	3.79	5.50	<0.001
	2008	2019	2.01	1.59	2.42	<0.001
Asian or Pacific Islander	2000	2007	1.06	−0.15	2.28	0.08
	2007	2015	−1.84	−2.83	−0.84	0.002
	2015	2019	−5.80	−7.91	−3.64	<0.001
White	2000	2009	5.33	4.87	5.78	<0.001
	2009	2014	3.32	2.09	4.57	<0.001
	2014	2019	−1.14	−1.91	−0.35	0.008
Hispanic	2000	2006	4.94	3.10	6.81	<0.001
	2006	2014	2.32	1.29	3.37	<0.001
	2014	2019	−1.71	−3.09	−0.31	0.02
Black	2000	2009	6.37	5.28	7.48	<0.001
	2009	2016	1.28	−0.13	2.71	0.071
	2016	2019	−6.07	−9.76	−2.22	0.005
American Indian/Alaska Native	2000	2019	3.99	2.48	5.52	<0.001

This pattern of rising and then declining HCC incidence was seen in most demographic strata that we examined with the notable exceptions of older individuals and the American Indian/Alaska Native population. In men, incidence rose during 2000–2009 (APC: 5.18%; 95% CI: 0.99 to 3.00) and during 2009–2015 (APC: 1.99%; 95% CI: 0.99 to 3.00) before falling from 2015 to 2019 (APC: −2.26%; 95% CI: −3.52 to −0.97). For women, HCC incidence began to decline a year earlier than in men (APC: 2014–2019, −1.40%; 95% CI: −2.44 to −0.35; Figure 1B). Rates began to fall in 2011 for those ages 20–54 years (APC: 2011–2019, −6.02%; 95% CI: −7.13 to −4.90) and in 2014 for those 55–64 years (APC: 2014–2019, −5.86%; 95% CI: −7.11 to −4.59). HCC incidence never fell among those ages 65–74 years but did reach a plateau during 2016–2019 (APC: 0.91%; 95% CI: −0.74 to 2.60). For those 75+ years of age, HCC incidence continued to climb throughout the study period, although at a slower pace during 2008–2019 (APC: 2.01%; 95% CI: 1.59 to 2.42) compared to 2000–2008 (APC: 4.64%; 95% CI: 3.79 to 5.50; Figure 1C; Table 2).

The year when rates peaked differed markedly by racial/ethnic group (Figure 1D). Rates among Asian/Pacific Islander individuals started to decline in 2007 (APC: 2007–2015, −1.84%; 95% CI: −2.83 to −0.84) and decreased more rapidly in the later years of the analysis (APC: 2015–2019, −5.80%; 95% CI: −7.91 to −3.64). In 2014, rates began to fall in the White (APC: 2014–2019, −1.14%; 95% CI: −1.891 to −0.35) and Hispanic populations (APC: 2014–2019, −1.71%; 95% CI: −3.09 to −0.31). 2 years later, rates began to fall in the Black population (APC: 2016–2019, −6.07%; 95% CI: −9.76 to −2.22). Among the American Indian/Alaska Native population, the highest annual HCC incidence was in 2017 (22.69), with lower rates in 2018 (16.87) and 2019 (19.64); however, there was no statistically significant change in the overall trend (APC 2000–2019: 3.99%; 95% CI, 2.48 to 5.52).

From 2000 to 2019, rates rose by 88% in American Indian/Alaska Native, 73% in White individuals, 52% in Black individuals, and 49% in Hispanic individuals, while falling by 22% in Asian/Pacific Islander individuals (Table 3). Over the entire two decades, rates were consistently and notably lower among White individuals. In 2019, compared to the White population, rates were more than twice as high among the American Indian/Alaska Native and Hispanic populations (incidence rate ratio [IRR], 2.83 and 2.23, respectively) and > 50% higher in the API (IRR, 1.74) and Black (IRR, 1.55) populations (Table 3).

**TABLE 3:** Incidence rate (per 100,000 persons), change in incidence, incidence rate ratio (IRR), and accompanying 95% confidence interval (95% CI) for hepatocellular carcinoma among those aged ≥20 years, by race/ethnicity—SEER 22, 2000–2019.

Race/ethnicity*	Incidence rate		Change in incidence (2000–2019)	IRR (95% CI)**	
	2000	2019		2000	2019
White	4.00	6.94	+74%	1.00	1.00
Black	7.05	10.74	+52%	1.76 (1.61–1.93)	1.55 (1.46–1.64)
Asian/Pacific Islander	15.59	12.11	−22%	3.90 (3.58–4.23)	1.75 (1.65–1.85)
Hispanic (all races)	10.43	15.49	+48%	2.60 (2.41–2.82)	2.23 (2.14–2.33)
American Indian/Alaska Native	10.47	19.64	+88%	2.61 (1.68–3.88)	2.83 (2.25–3.52)

* Individuals of Hispanic ethnicity were classified as ‘Hispanic’ and excluded from other race/ethnicity categories.

** The White population is the referent for IRR calculations.

## 4 DISCUSSION

Our analysis of SEER‐22 data during the years 2000 through 2019 shows that overall US HCC incidence peaked in 2015. HCC incidence started to decline in the Black population in 2016, 2 years after White and Hispanic populations and 7 years later than the Asian and Pacific Islander population. With that important finding, there is evidence for a significant downward trend in all US racial ethnic groups except the American Indian/Alaska Native population, and even among American Indian/Alaska Native individuals there was some indication for a decline in rates beginning in 2017. While these declines are encouraging, there continued to be wide disparities in HCC incidence; in 2019, HCC was more than twice as frequent in American Indian/Alaska Natives and Hispanic populations and >50% more frequent in Black individuals and Asian/Pacific Islanders compared to White individuals.

Several groups of investigators have examined recent data from SEER to determine the year of peak incidence for HCC in the United States. Previously, using data from the SEER‐21 registries through 2017, we found that HCC incidence peaked in 2014.^3^, ^4^ In contrast, based on data from 13 SEER registries for the years 1992–2017, Han et al. reported that HCC incidence began to decrease in 2011.^5^ Alvarez et al. also used SEER‐13 data, but extended the analysis to 2018, reporting that overall HCC incidence significantly declined between 2015 and 2018.^6^ The reason for those seemingly discrepant results is not clear. Now, using data from the largest grouping of SEER registries to date (i.e., SEER‐22), we also find that HCC incidence peaked in 2015. Some differences in estimates of the peak for HCC incidence in the United States may reflect use of different groupings of SEER registries, while other differences are more difficult to explain. Taken together, these reports provide evidence that HCC incidence in the United States began to decline in about 2015.

The most common causes of HCC in the United States are chronic hepatitis B (CHB), chronic hepatitis C (CHC), alcoholic liver disease (ALD), and nonalcoholic fatty liver disease (NAFLD). Of these factors, ALD and NAFLD are more common, but viral hepatitis infection carries a higher risk for HCC.^7^ SEER does not collect information on the presence of these etiologic factors or their treatment; however, data from other sources indicates that the prevalence of these conditions varies markedly by race/ethnicity. CHB is endemic in areas of Asia and Africa.^8^ In the 2011–2016 National Health and Nutrition Examination Survey (NHANES), CHB was most frequent in foreign‐born Asian (3.85%) and Black individuals (1.94%); prevalence was considerably lower in the US‐born population but greater in Asian (0.79%) or Black (0.52%) individuals compared to White individuals (0.08%).^9^ NHANES underestimates the true prevalence of HCV infection in the United States because some groups who at increased risk of CHC are underrepresented or excluded from the survey (e.g., people who inject drugs and people who are incarcerated).^10^ Nonetheless, it is noteworthy that the prevalence of CHC in 2011–2016 NHANES differed markedly by race/ethnicity: Black, 1.52%; Hispanic, 0.94%; White, 0.65%; Asian, 0.23%.^11^ These results from NHANES are consistent with our previous findings regarding the prevalence of viral hepatitis among older Americans with HCC, in which the Asian population had the highest incidence of HBV‐associated HCC and the highest incidence of HCV‐associated HCC was seen in the Black population.^12^ American Indian/Alaska Native are not well represented in NHANES, but other studies suggest a high prevalence of viral hepatitis in that group.^13^, ^14^ The frequency of ALD and NAFLD also vary by race/ethnicity. In a 2014 national survey, the frequency of heavy drinking (i.e., consuming ≥5 drinks on ≥5 days in the past month) in US populations was: American Indian, 9.2%; White, 7.1%; Hispanic, 5.1%; Black, 4.5%; Native Hawaiian/Pacific Islander, 4.6%; Asian, 2.0%.^15^ The frequency of NAFLD in population‐based cohorts was 22.9% in Hispanic individuals, 14.4% in the White population, and 13.0% in Black persons.^16^ The marked racial/ethnic differences in HCC incidence we observed likely reflect these differences in the prevalence of HCC risk factors.

Major improvements in the treatment of viral hepatitis occurred during 2000–2019. As neither alcohol use nor obesity decreased in the United States during that period,^17^, ^18^ more effective antiviral therapies likely explain the fall in HCC incidence. The effect of those advances in specific racial/ethnic populations depends on infection prevalence and treatment availability. Oral therapies that markedly reduce the risk of HCC in patients with CHB were introduced in 2005–2006,^19^ the years just prior to peak HCC incidence in the Asian/Pacific Islander population. Through most of the study period, treatment for CHC consisted of poorly tolerated interferon‐based regiments that resolved infection in ~40% of patients.^20^ In 2014, direct acting antiviral (DAA) therapies that raised the cure rate above 95% were introduced. Access to DAA regimens is lower among Medicaid recipients,^21^ who are disproportionately Black or Hispanic.^22^ Major limitations in the availability of DAA therapies through the Indian Health Service, the agency that provides medical care to the American Indian and Alaska Native populations, have been noted.^23^ We speculate that the introduction of DAA therapies in 2014, possibly in conjunction with the 2012 CDC recommendation for the HCV screening of all individuals born in the United States between 1945 and 1965,^24^ contributed to the peaking of HCC incidence the following year and that the later decline in HCC incidence in the Black and American Indian/Alaska Native populations reflects more limited access to DAA treatments. That scenario would point to the need for especially strong efforts to make treatment for hepatitis virus infections available to those populations.

A strength of this study is that SEER employs a rigorous protocol for diagnosis of cancer based on microscopic findings from pathology or cytology, radiography or clinical records and this paper presents results obtained from the largest collection of SEER registries that have been available to date. Our study also has important limitations. SEER does not collect information on etiologic factors that can cause HCC or on foreign nativity. We also lacked the information needed to examine subpopulations within the broadly defined Hispanic and Asian populations.

In summary, our study provides welcome evidence of a broad decrease in HCC incidence in the United States, but much remains to be done. Epidemiologically, it will be important to confirm that these trends continue in subsequent years. Efforts to identify and treat individuals with chronic viral hepatitis infections, especially in populations with a high prevalence of infection, as well as those with or at risk for ALD or NAFLD, are essential to ensure that trends in HCC incidence continue to improve.

## AUTHOR CONTRIBUTIONS

**Thomas O'Brien:** Conceptualization (lead); formal analysis (equal); methodology (supporting); writing – original draft (lead); writing – review and editing (equal). **Susan S. Devesa:** Conceptualization (equal); formal analysis (equal); methodology (equal); writing – review and editing (supporting). **Jill Koshiol:** Conceptualization (supporting); writing – review and editing (supporting). **Jorge Marrero:** Conceptualization (supporting); writing – review and editing (supporting). **Meredith Shiels:** Conceptualization (equal); formal analysis (lead); methodology (lead); writing – original draft (equal); writing – review and editing (equal).

## FUNDING INFORMATION

This work was supported by the Intramural Research Program of the National Institutes of Health, National Cancer Institute, Division of Cancer Epidemiology and Genetics. The content of this publication does not necessarily reflect the views or policies of the Department of Health and Human Services, nor does mention of trade names, commercial products, or organizations imply endorsement by the U.S. government.

## CONFLICT OF INTEREST STATEMENT

No conflict of interest exists for any of the authors.

## ETHICS STATEMENT

This study was exempt from Institutional Review Board review.

## Data Availability

The data used in this analysis may be requested from the National Cancer Institute, Surveillance, Epidemiology, and End Results Program https://seer.cancer.gov/data/access.html
